# Influence of the use of External Carbon Fiber Reinforcement on the Flexural Behavior of Prismatic Concrete Test Specimens. An Application for Repairing of Deteriorated Agricultural Structures

**DOI:** 10.3390/ma12121894

**Published:** 2019-06-12

**Authors:** Andrés Juan-Valdés, Desirée Rodríguez-Robles, Julia García-González, Manuel Ignacio Guerra-Romero, Julia Mͣ Morán-del Pozo

**Affiliations:** 1 Department of Agricultural Engineering and Sciences, University of Leon, Av. Portugal 41, 24071 Leon, Spain; julia.garcia@unileon.es (J.G.-G.); ignacio.guerra@unileon.es (M.I.G.-R.); julia.moran@unileon.es (J.M.M.-P.); 2 Department of Agronomy and Forestry Engineering, University of Extremadura. Av. Adolfo Suárez s/n, 06007 Badajoz, Spain; desireerodriguez@unex.es

**Keywords:** carbon fiber, concrete, agricultural buildings, flexural strength, laminates, surface treatment

## Abstract

This manuscript reports a study of the capacity of polymer composites to increase flexural strength in concrete. The polymer composites reinforced with carbon fiber and bonded with epoxy adhesive were used in prismatic test specimens of mass concrete corresponding to two different morphologies. The aim was to simulate the restoration of deteriorating concrete agricultural structures in order to explore the viability of this alternative against replacing them. An increase was found in the strength of the elements tested, with a higher strength being observed in those test specimens presenting a modified geometry.

## 1. Introduction

The passage of time, the weather conditions, and the action of various forces contribute to the degradation and loss of function of civil infrastructures and structures. There are two possible approaches to address this problem: The defective element can be replaced by a new one or it can be restored by incorporating some kind of reinforcement, so that the element remains functional. Given the current economic situation, the second option is preferable since it represents a cheaper and faster solution. However, the reinforcement requires a new knowledge about the materials needed to carry out the effective restoration properly.

The search for alternative techniques and new materials in this field has attracted the attention of many researchers and organizations interested in restoring damaged and defective structures. One of the most popular techniques used to improve the behavior of reinforced concrete elements has been the use of steel plates bonded with epoxy to the outer surfaces of beams and slabs. This technique is simple and effective in terms of cost and mechanical efficiency, but it has several disadvantages such as the corrosion of the steel plates with the consequent deterioration of the bond, the difficulty in handling heavy steel plates in construction works with restricted space, as well as limitations regarding the available length of the plate, which results in the need for joints [1,2]. Another commonly employed technique is to strengthen the structures through the construction of reinforced concrete jackets around the existing elements. Such a coating is clearly very effective in terms of strength, rigidity, and ductility, but it involves very intensive labor and creates unwanted weight. The jackets may also be made of steel but, in this case, protecting them from corrosion becomes a major problem [1].

In an attempt to improve on the aforementioned techniques, it is proposed to substitute the reinforcing materials cited above for fiber-reinforced polymer composites (FRP) as externally bonded (EB) laminate or as near surface-mounted (NSM) strip embedded into an indentation in the element. These composites have interesting structural characteristics, including immunity to corrosion, low weight (about one-quarter of that of steel, which facilitates their application in confined spaces and reduces labor costs), high voltage resistance, rigidity (which can be adapted to the design requirements), and a large deformation capacity. They are also available in a virtually unlimited range of sizes and geometries. Perhaps the greatest advantage of FRP is their adaptability. Depending on the specific load conditions of each element to be treated, the polymer can be positioned and applied in the most appropriate way to optimize performance [3]. Nevertheless, FRP also present certain disadvantages that should not be overlooked by engineers. Unlike steel, which behaves in an elastic–plastic manner, composite materials generally present an elastic fracture behavior, thus reducing ductility. Furthermore, weight for weight, the cost of the FRP materials is several times higher than that of steel [1].

The practice of strengthening concrete structures by bonding polymeric composites began in the 1990s in some Western European countries and in the USA. [3,4,5,6]. It is a very common method of improving the flexural strength of structures, but it is also normal to use this method as reinforcement against compression and shearing [7].

There are three main types of fibers used to strengthen civil engineering structures. These are glass, aramid, and carbon fibers. It should be noted that their physical and mechanical properties vary greatly from one type of fiber to another [1]. These composites consist of a large number of small, continuous, unidirectional non-metallic fibers with advanced characteristics, held together in a resin matrix. In the present study, a carbon-fiber-reinforced polymer (CFRP) was used. The method for applying these fibers to the existing concrete elements is to bond the positioned fiber externally by means of an epoxy adhesive system. The purpose of the adhesive is to provide a load path between the concrete surface and the composite material. Hence, one of the most important aspects to consider in order to achieve an effective reinforcement is a good bond between the fiber and the element being reinforced, since the mechanical performance and durability of FRP applications depend primarily on the bond with the structure [8]. It is only by integrating the carbon fiber and the reinforced element that an efficient use of the fiber is ensured.

In the last decades, various aspects of the laminated carbon fiber as a mean to retrofit concrete elements (such as various degrees of strengthening, durability, debonding, peeling, cracking, failure diagrams and compression, flexural and shear behavior of the reinforced element) have been studied [3,5,6,8,9,10,11,12,13,14,15,16,17,18,19,20,21,22,23,24,25,26,27,28,29,30,31]. These research efforts have led to the demonstration of the effectiveness of the composite materials as external reinforcing agents for strengthening concrete structures. Nonetheless, the selection of the materials used for strengthening different systems is a crucial process. Each system is unique in the sense that the fibers and resin components are designed to work together. The different systems correspond to different manufacturers and suppliers and are based on different configurations, types of fibers, adhesives, etc. In addition, the suitability of each system depends on the type of structure that must be restored [1].

**Figure 1:**
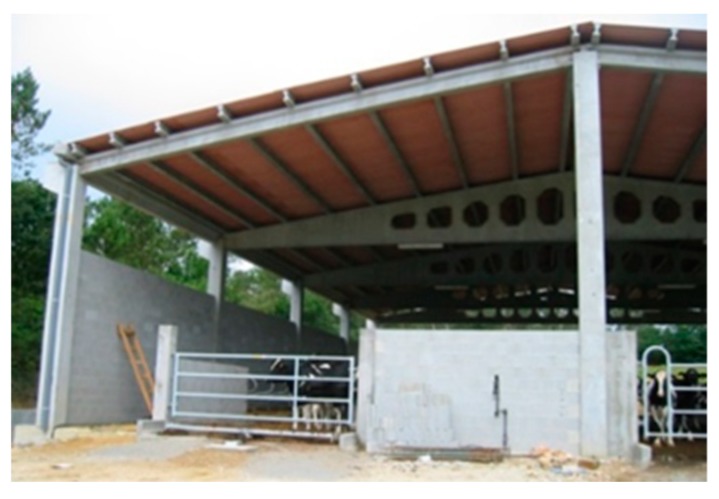
A typical concrete dairy farm showing pillars and beams.

One of the most common examples of buildings subjected to high levels of deterioration during their lifespan, especially from corrosion, are agricultural buildings. In most cases, these buildings are made of reinforced concrete and formed by frame structures of pillars and beams (Figure 1). Usually, the main stresses of these type of structures come from bending and not from compression, due to the relatively light weight of the different construction elements such as the roof.

**Figure 2:**
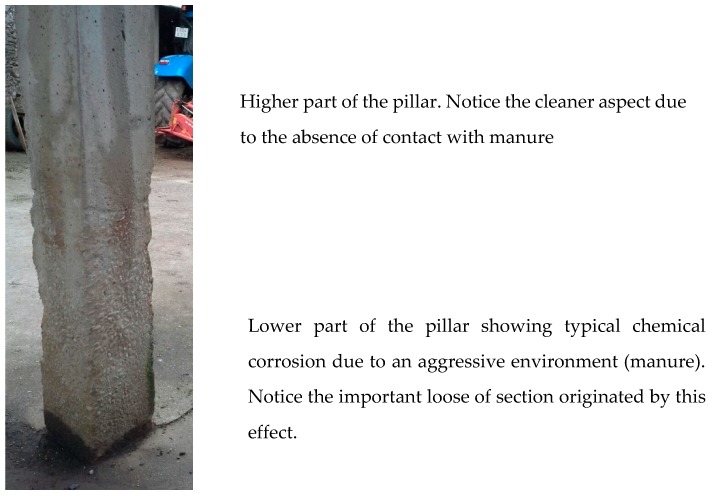
An image of a pillar showing typical deterioration by aggressive environment. Notice the loose of section in the lower part.

Concrete is unquestionably one of the most frequently used material for agricultural buildings, floors, and manure and silage storage structures [32]. In agricultural buildings, several cases have been studied [33,34] of concrete structures affected by corrosion. Most of these structures, especially the lower part of pillars (Figure 2), are exposed to erosion and/or chemical corrosion coming from the aggressive environment around them [33,34,35]. Among others, the presence of manure, acid pH, lactic and acetic acid, biogenic sulphuric acid, and chloride ion can provoke deterioration by corrosion and, in some cases, it is very severe [33]. Furthermore, since many of these structures are slender and relatively lightweight, they mainly receive important flexural stresses (when compared to the compressive stress) depending on the direction of the causal force (wind, asymmetric loads or design, different ice/snow accumulation, and traction cables). Thus, one of the faces of the bars will be subjected to a greater magnitude of traction than the other which, in addition to a possible corrosive situation, may cause the deterioration of the structure. Thus, the demand for a high-quality and durable concrete for these structures is essential. In this regard, some requirements are set forth in the different standards and codes and include the need for specific proportions of raw materials in the mixture, the use of special types of cement [36] (such as sulphate resistant cement), the use of additions, or the application of protective layers.

**Figure 3:**
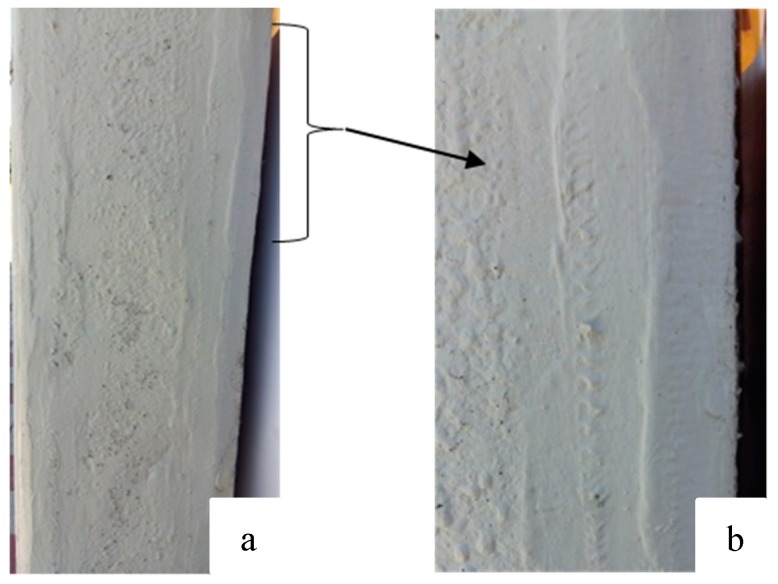
(**a**) Reparation of a pillar using carbon-fiber-reinforced polymer (CFRP). (**b**) Detail of the fibers.

The aim of this study is to determine whether the use of reinforcement composites based on carbon fiber is a suitable alternative for restoration of concrete bars presenting structural damage, caused by a mixture of corrosion, and bending originated by asymmetrical forces, which are common occurring conditions in agricultural settings. For these cases, the proposed restoration method can be an effective, inexpensive, and fast approach to solve the problem (Figure 3).

## 2. Materials and Methods 

### 2.1. Materials

#### 2.1.1. Concrete

The mix design for the concrete used in the tests was based on a soft consistency concrete with a characteristic compressive strength of 30 N/mm^2^. The proportions of the components were as follows in Table 1:

The strength, W/C ratio, and the rest of components were selected according to the Spanish Code on Structural Concrete [37] for an exposure class of high humidity (which entails an average annual rainfall over 600 mm) and XC3 according to Eurocode EN -1992 [38].

#### 2.1.2. Carbon Fiber Reinforcement

Reinforcement of the prismatic test specimens was carried out using two products from the Sika Company (Baar, Switzerland). Specifically, the fibers used were Sika Wrap-230 c/45 and the impregnation material used was Sikadur 330, whose mechanical properties can be seen in the Table 2.

### 2.2. Preparation of the Samples

As indicated in the manufacturer’s specifications, an important aspect that influences the effectiveness of the products is the absence of sharp edges on the substrate to receive the application, and, thus, it is recommended that the corners of the structure are rounded to a radius of at least 10 mm. Consequently, test specimens with blunt (chamfered) corners and test specimens with sharp edges were used in this study; firstly, to comply with the fiber application requirement and also to assess the differences in the fiber performance. Moreover, most of precast concrete pieces use chamfer strips to obtain chamfered corners; so, this point is studied in this work.

Following the protocol described in [37], two batches of concrete were made. One mixture was used to make standard prismatic test specimens and the other one was used to make the modified prismatic test specimens (with chamfered edges obtained by employing timber stops in the manufacture molds). Furthermore, in each batch, cylindrical test specimens were made in order to test the average characteristic strength of the resulting concrete. The experimental design of this assay was carried on as shown in Table 3.

Once the test specimens had been made and following 28 days of curing in water at 20 ± 2 °C, they were reinforced with SikaWrap-230 c/45 and Sikadur 330. 

As mentioned beforehand, when applying laminated fiber reinforcement, the pre-treatment of the element to be reinforced is of great importance in the effectiveness of both the adhesive and the bond formed with the CFRP sheet. Consequently, preparation of the test specimens included the drying of the concrete (moisture content less than 4 wt %) and sanding the rough surface with an angle grinder.

Regarding the preparation of the products themselves, the SikaWrap-230 c/45 fabric was cut to the desired size with scissors, taking care not to fold it as this would damage its properties. The resin and hardener (the two Sikadur-330 adhesive components) were initially stirred separately. They were mixed together in the proportions indicated (4:1) and stirred for 3 min until a uniform grey color was obtained. Then, the mixture was stirred slowly for an additional minute to remove any excess air.

**Figure 4:**
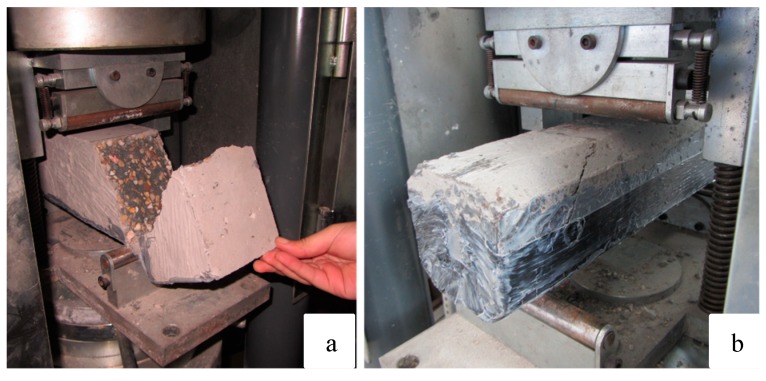
(**a**) Standard prismatic test specimens with reinforcement on the lower face. (**b**) Modified prismatic test specimen with reinforced lower face, chamfers, and side walls.

The Sikadur-330 resin mixture was applied differently depending on whether standard prismatic or modified prismatic test specimens were being used. In the first case, the fiber was placed on the surface of the lower face, whereas in the second case, the fiber was placed on the lower face and continued over the blunt corners to end halfway up the lateral faces of both sides (Figure 4).

Depending on the roughness of the substrate, between 0.7 to 1.2 kg/m^2^ of resin was applied to the concrete test specimens with a spatula until all empty spaces and uneven areas were filled. Subsequently, the SikaWrap-230 c/45 fabric was placed over the resin in the correct direction (i.e., with the fibers oriented parallel to the main axis of the prismatic test specimen). Lastly, in order to achieve a homogeneous surface, approximately 0.5 kg/m^2^ of resin applied as an additional and final coating. 

Curing was completed within a week since the process was carried out at a temperature above 23 °C. However, the test specimens were not subjected to loads until nine days after the CFRP application. It should be noted that, even so, there was an appreciable degree of adhesion on contact with the bare hands.

### 2.3. Methodology

#### 2.3.1. Compression Tests

In order to characterize the concrete used in the subsequent flexural tests, compression tests were initially conducted in order to verify that the concrete had reached the required strength in accordance with EN 12390-3:2009+AC:2011 [39]. For these tests, 150 × 300 mm cylindrical test specimens were used.

#### 2.3.2. Flexural Strength Tests

Two types of carbon-fiber-reinforced test specimens were subjected to flexural tests: The 100 × 100 × 400 mm standard prismatic specimens and the 100 × 100 × 400 mm modified prismatic specimens with longitudinal chamfered corners. Half of the specimens tested from each batch were coated with the CFRP whereas the other half remained uncoated and were used as control specimens.

In order to carry out the three point flexural test, the EN 12390-5:2009 [40] standard was followed in the study of both test specimen morphologies, even though fiber-reinforced specimens lack a formal test protocol. 

## 3. Results and Discussion

### 3.1. Compression Tests

Table 4 shows the results obtained in the compression tests. The results confirmed that the concrete from both batches met the initial requirement of a characteristic strength of 30 MPa.

### 3.2. Flexural Strength Tests

**Figure 5:**
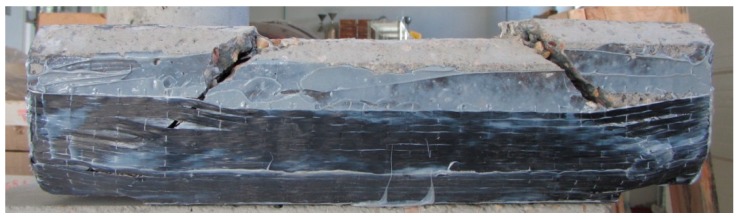
Fracture pattern of the prismatic specimen.

Several studies show the direct effect between a good concrete/CFRP bond and the increase of resistance of the repaired element [10,12,15]. Thus, special attention was paid to the mode of failure of the specimens. Fracture was gradual but ended with a sudden explosive noise. The assay was characterized by the crushing of the concrete followed by the break of the CFRP sheets at the point where the specimen had fractured, which in all cases was close to the rigid point support of the approved device (Figure 5). Subsequent inspection of the samples showed a fracture with good contact between the fiber sheet and the concrete, which indicates that the two elements had not separated at any time during the entire testing process.

**Figure 6:**
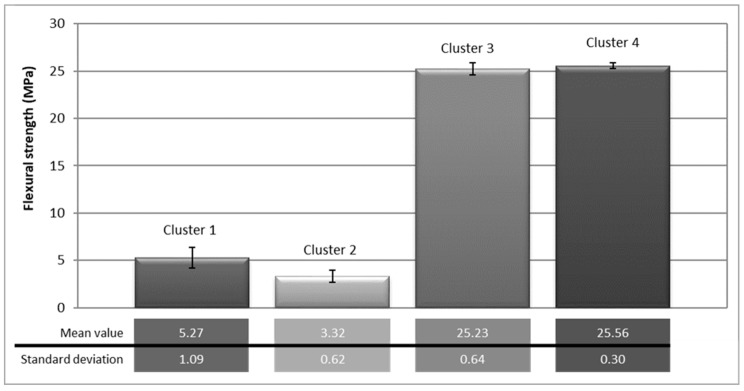
Flexural strength results by cluster.

In order to make a statistical analysis of the data, the results obtained in the flexural test of all specimens were gathered in four clusters bearing in mind the geometry and the presence/lack of reinforcement: Standard specimen without reinforcement (cluster 1), modified specimen without reinforcement (cluster 2), standard specimen with reinforcement (cluster 3), and modified specimen with reinforcement (cluster 4). Figure 6 shows the average flexural strength values for each defined group as well as the standard deviations calculated for each set of values.

As seen in Figure 6, reinforced specimens—both standard and modified—acquired a considerable increase in flexural strength compared to non-reinforced specimens. Since this is the phenomenon sought in this work, the analysis of the strength increase exhibited for both geometries was conducted. Table 5 shows the resistance increased (i.e., expressed as the improvement percentage) provided by the carbon fiber reinforcement for each group of samples with the same geometry.

Though, to date, very little amount of research has focused on the effect of this type of reinforcement in pillars under flexure efforts, some studies [13,41] have demonstrated the effectiveness of the use of composite materials as external reinforcement to strengthen concrete structures, and the work conducted by Alzate et al. [42] considered the external reinforcement with FRP sheets as a viable alternative to conventional methods in the rehabilitation of reinforced concrete structures. Quantitatively [43] demonstrated that the application of this type of reinforcement improved the static resistance up to 150%, while the results obtained in this paper show that the improvement reaches up to 379% for the standard specimens and up to 670% for modified specimens.

The different increase in the effectiveness of the reinforcement for both geometries tested is due to the better adhesion of the sheet in the chamfered edges of the modified specimens, which allowed the effective application of a greater amount of CFRP sheet.

However, despite the greater effectiveness of the reinforcement in the modified specimens, the statistical analysis showed that the final resistance values achieved were significantly similar for all the reinforced specimens. That said, there is no need to modify the edges of an existing structure in order to achieve better reinforcement results.

Although it had not been studied in the present article, the added effect that the use of CFRP laminates presents against corrosion should also be taken into account. For instance, the work conducted by Soudki and Sherwood [44] demonstrated the restoring ability of the carbon fibers in concrete beams damaged by corrosion with mass losses up to 15%.

## 4. Conclusions

In view of the previous results, the following conclusions can be drawn.

1. The rehabilitation method proposed in this paper is based on the reinforcement of concrete structures using carbon fiber sheets, which involves a resistance improvement from 379% to 670% depending on the type of edges presented by the element treated.
2. Reinforcement with carbon fiber sheets (SikaWrap 230 c/45 and Sikadur 330) provides improved flexural strength in prismatic test specimens.
3. It is of great importance to apply the CFRP sheets to the surface of the reinforced element correctly, since poor adhesion of the fiber will lead to a defective fracture. As the results obtained indicate, the desired level of reinforcement will not be achieved if there is a poor bond, due to the incomplete transfer of stress from the fiber sheet to the concrete. The results obtained in this article show a great improvement in the efficiency of the reinforcement, almost doubled, when applied on elements with chamfered edges instead of sharp edges.
4. The manufacturer’s recommendation to round the edges of the element to be treated before the application of the product could be disregarded since the increase of resistance achieved with a better concrete–CFRP bonding would have been previously decreased as a result of the reduction in the dimensions of the modified element.
5. The corrosion protection provided by this type of reinforcement is another positive aspect that renders it even more advisable in the retrofitting of damaged concrete structures, especially in agricultural environments.

## Figures and Tables

**Table 1:** Concrete mix design.

Proportions per Cubic Meter (kg)	
Characteristic strength (N/mm^2^)	30
Ratio water/cement	0.5
Portland cement (CEM III/A 42.5 N/SR, Cementos Tudela Veguin, Aboño, Spain)	312.5
Water	155.21
Sand 0/4 mm	96.98
Sand 0/5 mm	441.81
Gravel 4/10 mm	484.92
Gravel 6/12 mm	161.64

**Table 2:** CFRP laminates and epoxy adhesive properties.

**SikaWrap-230 C/45** **High-strength carbon fibers**	Fiber Orientation	0° (Unidirectional).
	Weight	225 g/m^2^
	Thickness	0.13 mm
	Tensile strength	3500 N/mm^2^
	Elasticity Modulus	230,000 N/mm^2^
	Fracture elongation	1.5%
**Sikadur 330** **Epoxy resin**	Tensile strength	30 N/mm^2^
	Flexural modulus	3800 N/mm^2^
	Tensile modulus	4500 N/mm^2^
	Fracture deformation	0.9%

**Table 3:** Experimental design.

Type of Test Specimens	Dimensions	Test	Standard
Cylindrical	150 × 300 mm^2^	Compression	EN 12390-3
Prismatic	100 × 100 × 400 mm^3^	Flexural	EN 12390-5
Chamfered Prismatic	100 × 100 × 400 mm^3^	Flexural	EN 12390-5

**Table 4:** Compressive characteristic strength for cylindrical test specimens (150 × 300 mm).

Samples	Compressive Characteristic Strength (MPa)
First batch	32.87 ± 1.45
Second batch	35.55 ± 2.22

**Table 5:** Improvement percentages provided by the carbon fiber reinforcement.

Specimen Geometry	Improvement (%)
Standard	379
Modified	670
